# Clinical Society of Maryland

**Published:** 1892-04

**Authors:** W. T. Watson

**Affiliations:** Secretary; 1603 N. Broadway, Baltimore, Md.


### *SOCIETY REPORTS.*

---

### CLINICAL SOCIETY OF MARYLAND.

---

BALTIMORE, February 19, 1892.

DR. HIRAM WOODS read a paper on "The Treatment of Granular Conjunctivitis," and exhibited patients treated by the method he now uses, viz., that of Dr. Knapp, which is the squeezing out of the spawn-like lymph follicles by means of forceps specially adapted to the purpose. Case 1, a man with pannus of three months' duration. Had been treated all this time with blue stone with no improvement. Forceps were used very gently; some granulations pressed out. Considerable pain and much bleeding from conjunctiva ensued. Following day man's eyes were wide open and photophobia completely relieved. Squeezed out some more granulations. He came back next day with conjunctiva quite clear. Has not returned since. Case 2, girl with so-called diffused trachoma of two years' duration. Was treated all last summer with blue stone. In November was suffering intensely, the entire upper lid of right eye covered with spawn-like, soft follicular granulations extending over into the ocular conjunctiva. Two operations performed. At the first nearly all the granulations of palpebral conjunctiva were squeezed out; at the second all those that had escaped in the first operation were destroyed. There was swelling and pain for a couple of days, then these symptoms disappeared and photophobia, also. There are still a few granulations in the retrotarsal fold which will be removed. The palpebral surface is quite smooth. Case 3, follicular trachoma of long duration. Granulations of connective tissue variety buried deep in conjunctiva. Dense, heavy pannus along upper part of cornea and whole cornea vascular. Follicles pressed out with exercise of considerable force. Conjunctiva became perfectly smooth. After a month inflammation was set up by small amount of jequirity with the view of clearing up the pannus. The pannus cleared up, the photophobia has entirely disappeared and the eye is almost well. Case 4, man troubled with trachoma for four years. Came to hospital early in January and was operated on without previous treatment. Granulations were of connective tissue kind, a great deal of thick heavy pannus. Photophobia considerable, lachrymation much, and whole eye congested. Granulations squeezed out by using considerable force. There was a good deal of pain and considerable reaction and after two weeks the eye was watery and somewhat painful. There is now no watering, the eye is clearing up, the lid is smooth and he is in a fair way to get well of his pannus. Two other cases, both in young Jewish women, were in the atrophic stage, and very little could be done except to relieve irritative symptoms. Both suffering intensely from photophobia and lachrymation. One operated on a week ago and is almost entirely relieved of photophobia. The other, operated on yesterday, feels better to-day than before the operation. Two cases operated upon at the hospital never returned.

His experience with these cases, together with the experience of Dr. Knapp in his 114 published cases, leads Dr. Woods to the conclusion that this is the best method ever devised for the relief of granular conjunctivitis.

DR. J. E. MICHAEL: I was for a number of years Dr. Chisolm's first assistant at the Presbyterian Eye and Ear Hospital, and I remember very vividly the many cases of trachoma that came to us day after day and month after month and year after year, to have nitrate of silver or blue stone applied, and it was our habit to regard these cases as almost hopeless. I have noticed, of course, a gradual improvement in some of them, which would go to a certain point and then stop. I have never seen any cases which have shown anything like the improvement seen in these cases exhibited by Dr. Woods. I want to express my satisfaction that so important an advance has been made in treating such an obstinate and troublesome pathological condition.

DR. WILMER BRINTON read a paper on "Phlegmasia Alba Dolens, with Report of Three Cases."

In about 1,100 cases of obstetrics Dr. Brinton has seen three cases of phlegmasia alba dolens, or the so-called "milk-leg." The various views as to its causation were given. It is now generally held that it is caused by phlebitis, that phlebitis being an extension of the disease from the vessels of the uterus. Virchow claims it to be due to a physiological thrombosis.

CASE I.—Mrs. D.; confined in November, 1884; second child; labor rapid; lying-in period uneventful until eleventh day; temperature and pulse normal for seven days, the record no longer kept. On eleventh day patient was found in bed, crying with pain in left leg. Pulse 120, temperature 101½. Had a chill in the morning, followed by feeling of *malaise* and intense pain in left leg. Leg was swollen and hot to touch. Swelling much greater next day. Pulse and temperature became normal in a few days. Swelling gradually disappeared, first from foot, then from the calf and then from the fleshy part of the leg. It was three months before she ceased to complain of stiffness and soreness.

CASE II.—Mrs. S., confined by a midwife October 8, 1888; no trouble or complications; remained in bed till tenth day, and then resumed her domestic duties. On the night of the fourteenth day after confinement had a chill, followed by pain through body and intense headache. Next morning was somewhat better but could not move left leg without pain, and it was rapidly swelling. Dr. Brinton was called in next day and found patient in bed, pulse 120, temperature 101½. Complained of a general feeling of *malaise*, severe headache and very severe pains in left leg. Leg much swollen and œdematous, especially in calf and about the ankle. Especially tender to touch on inner side of popliteal space. In two days swelling about ankle began to disappear. In seventeen days got out of bed and soon began to move about and attend to household duties.

CASE III.—Mrs. T., delivered September 1, 1891, of twins. It was a case of placenta prævia centralis, with much loss of blood, from which the patient rapidly recovered. Lying-in period uneventful although pulse and temperature slightly above normal. Pulse 85 to 100 and temperature 99½ to 101. On tenth day sat up for a short time. On evening of eleventh day temperature rose to 104 and pulse to 126. Had had decided rigor about midday. Next morning pulse 100, temperature 101. Examination revealed a case of septic endometritis, due, doubtless, to lacerated cervix. The "skilled" nurse had given the injections in such an imperfect manner that no benefit had been derived. On the fourth day from the beginning of the attack the left leg showed marked signs of phlebitis. Pain first felt below Poupart's ligament, and extending down the thigh to the leg. The leg became greatly swollen. In ten days painful symptoms subsided and patient moved the limb without much pain, when suddenly pulse became rapid, temperature 104, and right leg became involved more extensively than the left. About seven weeks from time of delivery she was able to be removed to Washington. She is now enjoying the best of health.

The treatment of these cases was by internal administration of quinine, opium, aconite and phenacetin, and locally absolute rest of limb, application of flaxseed poultices to certain parts of limb for a few days, and later the limb was rubbed from time to time with camphorated oil and a flannel bandage applied daily from toes upward. In Case III. the uterus was washed out daily for some time with bichloride solution.

DR. W. S. GARDNER: I would like to ask Dr. Brinton if he kept the temperature record of that first case up to the time she was attacked?

DR. BRINTON: I did not. It is now several years since but I am satisfied that the pulse and temperature were practically normal; if not I would have made a record of the case.

DR. GARDNER: There is quite a difference between a "practically" normal temperature and an actually normal temperature. I believe that if the temperature records of all these cases are kept accurately you will find that few, if any, will have a normal temperature from the time of confinement till the time that phlegmasia alba dolens comes on. I think it is a fact that is about as well established as anything connected with septic troubles of the puerperal state that this is one of the conditions that we have as the result of septic infection; that it is nothing more than a connective tissue inflammation in the leg due to sepsis. The clot in the veins is entirely a secondary affair and has nothing materially to do with the condition. There are many post mortems reported in which there were no clots and no phlebitis. Even if this were not the case the retarding of the return flow of blood would not give the condition found in phlegmasia alba dolens. Retarding would give you a simple œdema; in phlegmasia you have in addition to œdema what seems to be more of an inflammatory condition, although it is not associated with the redness of ordinary inflammation; it is an infiltration into the tissues instead of a pouring out of serous fluid into spaces beneath the skin, so that the limb becomes practically solid and does not pit readily on pressure.

So far as the treatment of these cases is concerned, it is just the treatment of all our infectious diseases except syphilis and malarial fever. You cannot do anything with them; they either get well or they die. You cannot cure typhoid fever, nor scarlet fever, nor phlegmasia alba dolens, nor troubles where there are micro-organisms developing in the tissues. You can only treat the symptoms as they arise.

DR. J. E. MICHAEL: The question as to the necessarily septic nature of phegmasia alba dolens is not by any means settled and Dr. Gardner's statement that a careful record would in all cases show a rise of temperature or other conditions indicating a septic state of the patient is not carried out by the facts in many instances. I am convinced that Dr. Brinton's cases are as he stated them to be. He took the temperature for a certain number of days, and finding no rise did not take it again. I have seen one case of phlegmasia. The woman was dropsical, badly nourished and badly cared for. She had general œdema and œdema of the lung and every evidence of advanced kidney disease. She was confined successfully. For several days her temperature was normal, that is under 100; for we regard, in such cases, anything under a hundred as normal. On the twelfth day there was the sudden occurrence of pain and the other symptoms which Dr. Brinton has given as indicative of beginning phlegmasia alba dolens, and the case turned out to be so and had a fatal issue. The uterus, vagina and everything connected with the generative organs were absolutely free from any evidence of previously existing inflammation. There was a clot in the femoral iliac vein which had undergone softening and was described by the pathologist as the puriform softening of Virchow. I think there is a question if there was anything which comes under the description of puerperal septicæmia and its various manifestations. What adds to the interest of this discussion is the statement made by Dr. Brinton, which is in accordance with our histories and experience, that cases of phlegmasia alba dolens occur, as a rule, in patients having a normal puerperium. The condition of the blood, or whatever it may be which predisposes it to easy clotting, is undoubtedly, according to the views of Virchow, responsible for its clotting under these circumstances. I am convinced that at least a portion of these cases are not associated with distinct phlebitis but are the result of primary clot formation and do not begin till the clot is formed.

The other side of the case, and one that is taken by a good many, including Dr. Gardner, is that phlegmasia alba dolens always indicates a septic condition. I am inclined to think that there is a septic condition which produces, clinically speaking, the same condition which we find in phlegmasia alba dolens. We have the occurrence of phlebitis in the neighborhood of the generative tract and in the adnexa, and we may have a phlebitis which would involve the general vein and would produce the clot and the general train of symptoms following. I do not think we have grounds for sepsis in all the cases. I believe the only satisfactory solution can be arrived at by gathering together all possible information about the occurrence of this disease in lying-in hospitals now and comparing it with its occurrence in years gone by when septic conditions prevailed to such an extent. Virchow says that in the examination of these clots in cases that terminate fatally we have an appearance of pus surrounding the vessel and which would on careless examination be taken for pus, but in which the most scrutinizing examination reveals no pus and no bacteria; so I am inclined to think that we can have phlegmasia alba dolens with no septic infection whatever.

I am inclined to think that two of the cases spoken of by Dr. Brinton were of this kind.

Another point bearing on this subject in a most practical way is that in by far the great majority of cases where we do have positive puerperal septicæmia we do not have phlegmasia alba dolens.

DR. J. H. BRANHAM: As Dr. Michael has said it is difficult to decide whether all cases of this disease have the same cause. Where there is a clot in the vein, if the trouble begins as an infection, the organisms enter some of the uterine vessels and gradually extend to the larger vessels; this is the theory maintained by many good observers. If it is simply clotting of blood extending from the smaller veins into the larger ones, this can occur without sepsis. Some cases are not accompanied by vein clots at all; in these cases the swelling is due, I think, to stoppage of the lymph vessels.

The occurrence of chill, and the rise of temperature and pulse in these cases look as though there was some form of septic infection. I do not believe that simple stoppage of circulation, without some infection in addition, causes these symytoms. As to how the infection gets there there is some doubt. It is well known that we may have a very late form of sepsis in obstetrical cases. Without any previous rise of temperature decided septic trouble may come on ten to eleven days after labor. Either there was a late infection or there was at first a very slight infection and then it took time for sufficient development of the organisms to produce decided septic symptoms.

DR. W. S. GARDNER: With reference to the history of these cases, we know that it is an extremely rare disease. Tyler Smith gives us a history of one man having three successive cases of labor in each of which phlegmasia alba dolens occurred. There was another series of three successive cases in this town a few years ago. While these series of cases are very short, and might be considered as coincidences in any ordinary disease, yet considering the extreme rarity of phlegmasia alba dolens, I think a series of even three cases is strong presumptive evidence that it must be due to something which can be communicated by some one to the patients.

If the remarkable degree of disorganization which is found in these cases is not due to micro-organisms, how then are you going to account for it?

DR. BRINTON: I am inclined to think that sepsis is the cause in a certain number but not in all cases.

As to normal temperature: In the vast majority of cases I think that the temperature will be 98°–99°, and that in many cases there is a normal temperature of 100.5°. In some cases where the temperature has been 100°, the lying-in period has been as uneventful as where the temperature was 98.5°.

W. T. WATSON, M. D., *Secretary.*

1603 *N. Broadway, Baltimore, Md.*

